# Prognostic Nutritional Index and a Blood-Based Prognostic Tool in Prostate Cancer Treated with Abiraterone, Enzalutamide or Cabazitaxel

**DOI:** 10.3390/medicina61061105

**Published:** 2025-06-18

**Authors:** Hakan Taban, Mustafa Erman, Deniz Can Guven, Burak Yasin Aktas, Feride Yilmaz, Serkan Yaşar, Hasan Cagri Yildirim, Ferit Aslan, Sercan Aksoy

**Affiliations:** 1 Medical Oncology Clinic, Medical Park Ankara Hospital, 06370 Ankara, Turkey; feritferhat21@gmail.com; 2 Department of Preventive Oncology, Hacettepe University Cancer Institute, 06230 Ankara, Turkey; ermanm1968@gmail.com; 3 Department of Medical Oncology, Hacettepe University Cancer Institute, 06230 Ankara, Turkey; denizcguven@hotmail.com (D.C.G.); byaktas@hotmail.com (B.Y.A.); saksoy07@yahoo.com (S.A.); 4 Medical Oncology Clinic, Samsun Education and Research Hospital, 55090 Samsun, Turkey; doktorferide@gmail.com; 5 Medical Oncology Clinic, Dr. Abdurrahman Yurtaslan Ankara Oncology Training and Research Hospital, 06200 Ankara, Turkey; dr.wetsiz@hotmail.com; 6 Medical Oncology Clinic, Nigde Omer Halisdemir University Training and Research Hospital, 51200 Nigde, Turkey; hasan-cagri@windowslive.com

**Keywords:** abiraterone, cabazitaxel, enzalutamide, prognostic nutritional index, prostate cancer

## Abstract

*Background and Objectives*: The prognostic nutritional index (PNI), a marker reflecting both nutritional and immune status, has been associated with prognosis in various malignancies. However, evidence in metastatic castration-resistant prostate cancer (mCRPC), particularly from non-Asian populations, remains limited. This study aimed to evaluate the prognostic value of baseline PNI and to develop a blood-based prognostic model in mCRPC patients treated with abiraterone acetate (AA), enzalutamide (ENZA), or cabazitaxel (CABA). *Materials and Methods*: This retrospective study included mCRPC patients treated with AA, ENZA, or CABA before or after docetaxel. PNI was calculated as: 10 × serum albumin (g/dL) + 0.005 × total lymphocyte count (/mm^3^). Patients were classified into low-PNI (≤40.8) and high-PNI (>40.8) groups using the median PNI value. Survival outcomes were analyzed using Kaplan–Meier and Cox regression methods. *Results*: A total of 299 patients were analyzed: 133 (44.5%) received AA, 106 (35.5%) ENZA, and 60 (20.0%) CABA. Patients with high PNI had significantly longer median overall survival (OS; 30.2 vs. 12.6 months, *p* < 0.001), radiologic progression-free survival (rPFS; 13.5 vs. 6.7 months, *p* < 0.001), and PSA progression-free survival (PSA-PFS; 10.2 vs. 5.1 months, *p* < 0.001). These associations remained significant across all treatment subgroups. In multivariate analysis, prostate surgery (HR: 0.6), high PNI (HR: 0.5), PSA response (HR: 0.5), and elevated ALP (HR: 1.6) were independent predictors of OS. A prognostic model incorporating PNI, alkaline phosphatase, and anemia stratified patients into four risk groups with distinct OS: 49.1, 30.8, 18.8, and 9.1 months, respectively. *Conclusions*: This is the largest study to date in a non-Asian mCRPC population showing that baseline PNI is a strong, independent prognostic factor for survival. The proposed blood-based tool may aid in clinical risk stratification, pending prospective validation.

## 1. Introduction

Prostate cancer is the second most common cancer after lung cancer and the 5th cause of cancer-related deaths in men worldwide [1], accounting for 7.3% of cancer-related deaths, with approximately 400,000 deaths annually [1]. Although most cases are diagnosed at a localized or locally advanced stage, approximately 8.2% are detected at an advanced stage [2]. According to U.S. data, the five-year overall survival (OS) rate for prostate cancer is 98% across all stages but drops to around 30% for patients diagnosed at an advanced stage [2].

Similarly to breast cancer, prostate cancer is highly responsive to anti-hormonal therapy. Androgen deprivation therapy (ADT) has long been the standard first-line treatment for metastatic prostate cancer. However, most patients eventually progress to a castration-resistant state. Adding docetaxel (DOCE) or one of the androgen-receptor signaling inhibitors (ARSIs) such as abiraterone acetate (AA) or enzalutamide (ENZA) to ADT is the current preferred treatment options in first-line therapy in metastatic castration-resistant prostate cancer (mCRPC) [3]. Abiraterone acetate is a selective CYP17 inhibitor that blocks androgen biosynthesis in the testes, adrenal glands, and prostate tumor tissue [4]. Enzalutamide, on the other hand, is a potent oral androgen receptor (AR) antagonist that inhibits androgen receptor nuclear translocation, DNA binding, and coactivator recruitment, effectively suppressing AR-mediated transcription [5]. Both agents have demonstrated significant overall survival benefits in randomized clinical trials and are frequently used in both pre- and post-chemotherapy settings [3]. Cabazitaxel (CABA) is a next-generation taxane that can be used in progression after DOCE in mCRPC [6]. In addition to these treatment options, poly (ADP-ribose) polymerase (PARP) inhibitors, PARP inhibitors plus ARSI combinations, radionuclide therapies, and immunotherapies are the next-line approved treatment options in selected patients [3].

Molecular prognostic biomarkers such as androgen receptor aberrations, PTEN loss, DNA repair gene deletions, TP53 mutations, and RB1 loss have been identified in mCRPC [7]. Besides these molecular alterations, increasing evidence suggests that systemic inflammatory response also plays a critical role in many solid tumors, including prostate cancer [8,9,10,11]. Previous studies have demonstrated that inflammatory markers such as neutrophil lymphocyte ratio (NLR), platelet lymphocyte ratio (PLR) and systemic immune inflammatory index (SII) may have prognostic value in prostate cancer [11,12]. In addition, the nutritional status of cancer patients has also been reported to influence treatment outcomes [8,13].

The prognostic nutritional index (PNI) is a simple and practical tool that reflects both nutritional and inflammatory status, calculated from serum albumin concentration and peripheral lymphocyte count. First described by Onodera et al., low PNI values were initially associated with poor prognosis in patients with gastrointestinal cancers [14]. In recent years, low PNI has also been linked to worse survival outcomes in several other malignancies [15,16,17,18,19,20,21]. While several studies have demonstrated the prognostic value of PNI in prostate cancer patients of Asian origin, data on non-Asian populations remain limited, with only a few small-scale studies exploring this association [9,10,22,23]. Therefore, the aim of this study was to evaluate the prognostic impact of baseline PNI on treatment response and survival in mCRPC patients treated with AA, ENZA, or CABA.

## 2. Materials and Methods

### 2.1. Study Design and Patient Population

This retrospective study included patients diagnosed with mCRPC who received AA, ENZA, or CABA before or after DOCE at Hacettepe University Cancer Institute between January 2011 and December 2020. Patients in the AA and ENZA groups were treated either prior to or following DOCE based on clinical status, physician discretion, and drug availability at the time of treatment, as both agents have FDA approvals for use in pre- and post-docetaxel settings. In contrast, all patients in the CABA group received cabazitaxel after progression on DOCE, in accordance with its FDA-approved indication for post-docetaxel mCRPC.

Demographic, pathological, and clinical data were collected from the hospital’s electronic medical records. Baseline laboratory parameters, including prostate-specific antigen (PSA), complete blood count, and biochemistry results were recorded. Anemia was defined as hemoglobin levels <13.5 g/dL, consistent with commonly used thresholds in the literature. Additionally, data regarding PSA and radiologic responses, progression dates, and dates of last follow-up or death were obtained.

### 2.2. Prognostic Nutritional Index Calculation

The prognostic nutritional index PNI was calculated using the following formula:

PNI = (0.005 × total lymphocyte count per mm^3^) + (10 × serum albumin in g/dL) (1)

This formula was first described by Onodera et al. [14].

PNI levels were calculated using laboratory values obtained within 1 to 7 days prior to the initiation of AA, ENZA, or CABA therapy.

### 2.3. Definition of Clinical Outcomes

The clinical endpoints evaluated in this study included overall survival (OS), radiographic progression-free survival (rPFS), and PSA progression-free survival (PSA-PFS). OS was defined as the time from the initiation of AA, ENZA, or CABA therapy to death from any cause or last follow-up. rPFS was defined as the interval between treatment initiation and the occurrence of radiological disease progression, death, or last follow-up. PSA-PFS was defined as the time from the start of treatment to PSA progression, death, or last follow-up whichever occurred first.

Radiographic progression was determined based on the Response Evaluation Criteria in Solid Tumors (RECIST) for nodal and visceral metastases, and the appearance of at least two new bone lesions on bone scintigraphy, according to the criteria established by the Prostate Cancer Working Group 2 (PCWG2) [24]. PSA progression was defined as a ≥25% increase in PSA from the nadir value (with an absolute increase of at least 2 ng/mL), confirmed by a second measurement obtained at least three weeks later. A PSA response was defined as a decline of ≥50% in PSA levels from baseline [24].

### 2.4. Statistical Analyses

Descriptive statistics were presented as median with interquartile range (IQR; 25th–75th percentile) or as mean ± standard deviation (SD) for continuous variables, and as frequencies and percentages for categorical variables. Comparisons between independent groups were performed using the independent samples t-test or the Mann–Whitney U test, depending on the distribution of variables. Categorical variables were compared using the Chi-square test.

To identify the optimal prognostic nutritional index (PNI) threshold, a receiver operating characteristic (ROC) curve analysis was conducted. A cut-off value of 41.26 was identified, yielding a sensitivity of 59.1%, a specificity of 59.7%, and an area under the curve (AUC) of 0.643, indicating a moderate discriminative performance. Additionally, since the median PNI value has been commonly used for patient stratification in previous studies, the median PNI in this cohort was also calculated and found to be 40.8. As no cut-off point with both high sensitivity and specificity could be determined, and the ROC-derived value was found to be very close to the median, patients were classified into low and high PNI groups based on the median value to ensure consistency and comparability with existing literature. Accordingly, the patients were classified based on the median PNI value as low PNI (PNI ≤ 40.8) and high PNI (PNI > 40.8).

Survival analyses were performed using the Kaplan–Meier method, and differences in survival between prognostic subgroups were compared using the log-rank test. Multivariate analyses were carried out using Cox proportional hazards regression (enter method), including variables that were statistically significant in univariate analyses. Hazard ratios (HRs) and 95% confidence intervals (CIs) were calculated. All statistical analyses were performed using IBM SPSS Statistics version 22. A two-sided *p*-value of <0.05 was considered statistically significant.

## 3. Results

### 3.1. Patient Characteristics

A total of 299 patients with mCRPC were included in the study. The mean age at prostate cancer diagnosis was 65.2 years (±9.0). More than half of the patients (59.9%) presented with metastatic disease at diagnosis, and the majority (78.9%) had a Gleason score of 8 or higher. Radical prostatectomy was performed in 20.4% of patients, and 27.1% had a history of definitive or salvage radiotherapy to the primary tumor. Treatment distribution was as follows: 133 patients (44.5%) received AA, 106 (35.5%) received ENZA, and 60 (20.0%) were treated with CABA. Additional baseline clinicopathologic characteristics are summarized in Table 1. The detailed baseline characteristics of each treatment subgroup are provided in Supplementary Table S1.

The median calculated PNI was 40.8. PNI was ≤40.8 in 148 patients (49.5%) and >40.8 in 151 patients (50.5%). Patients in the low PNI group were older (*p* < 0.001), had higher baseline PSA levels (*p* < 0.001), were more frequently anemic (*p* < 0.001), and exhibited lower serum albumin (*p* < 0.001), higher alkaline phosphatase (ALP) (*p* < 0.001), and elevated lactate dehydrogenase (LDH) levels (*p* = 0.001) compared to patients with high PNI. The other baseline characteristics were similar across the low and high PNI groups, Table 2. Pre-treatment clinicopathological characteristics according to treatment subgroups are summarized in Supplementary Table S2.

### 3.2. Overall Survival, Radiological Progression-Free Survival and PSA Response Analysis

The median follow-up duration for the entire cohort was 18.9 months (interquartile range [IQR], 9.3–30.2). The mOS was 20.7 months (95% CI, 17.0–24.4) in the AA group, 24.1 months (95% CI, 19.8–28.6) in the ENZA group, and 16.1 months (95% CI, 9.4–22.8) in the CABA. Overall survival outcomes according to treatment subgroup and docetaxel treatment sequence are presented in Supplementary Table S3.

Regarding rPFS, the median rPFS was 10.1 months (95% CI, 7.6–12.4) in the AA group, 11.4 months (95% CI, 7.6–15.2) in the ENZA group, and 7.6 months (95% CI, 6.0–9.1) in the CABA group. Radiologic progression-free survival outcomes according to treatment subgroup and docetaxel treatment sequence are presented in Supplementary Table S4.

PSA response was evaluable in 282 patients (94.3%). Among them, 58.2% demonstrated a PSA response, defined as a ≥50% decline from baseline PSA levels. The mOS was significantly longer in patients who achieved a PSA response compared to those who did not (29.8 months [95% CI, 25.4–34.2] vs. 12.1 months [95% CI, 9.3–14.9]; *p* < 0.001).

### 3.3. Survival Analysis According to Prognostic Nutritional Index

**Figure 1:**
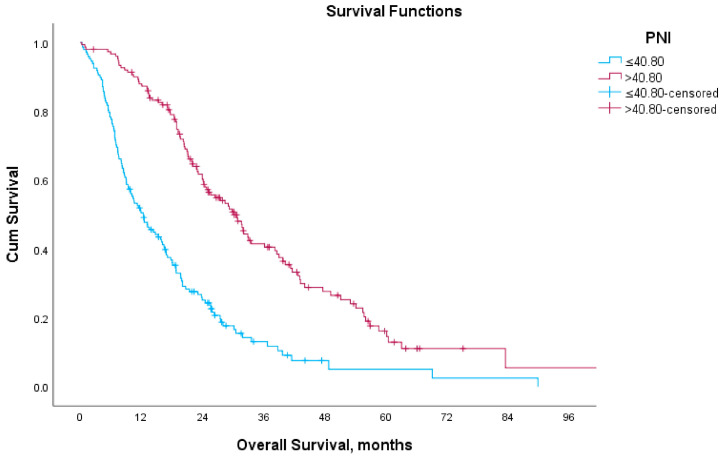
Kaplan–Meier curves for overall survival according to PNI.

**Figure 2:**
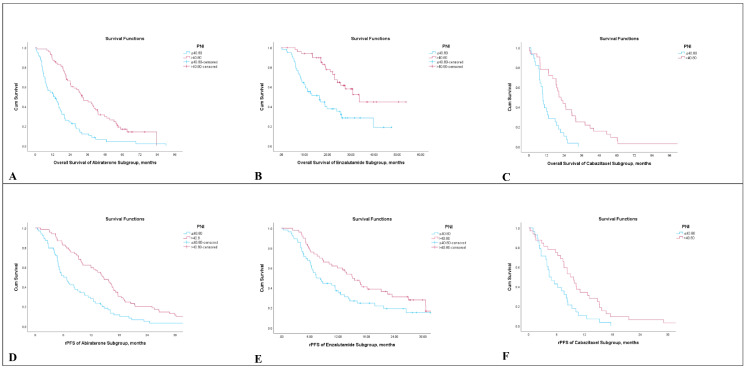
Kaplan–Meier curves of the subgroups according to PNI. (**A**), overall survival of abiraterone acetate subgroup. (**B**), overall survival of enzalutamide subgroup. (**C**), overall survival of cabazitaxel subgroup. (**D**), progression-free survival of abiraterone acetate subgroup. (**E**), progression-free survival of enzalutamide subgroup. (**F**), progression-free survival of cabazitaxel subgroup.

The mOS was 12.6 months (95% CI; 9.1–15.6) in the low-PNI group and 30.2 months (95% CI; 25.4–35.0) in the high-PNI group, (Figure 1). When stratified by treatment modality, patients treated with AA had a mOS of 12.5 months (95% CI, 6.0–19.0) in the low-PNI group and 32.1 months (95% CI, 25.1–39.1) in the high-PNI group. For ENZA, the mOS was 16.5 months (95% CI, 9.5–23.4) and 33.6 months (95% CI, 24.2–43.0) in the low- and high-PNI groups, respectively. In patients treated with CABA, mOS was 9.4 months (95% CI, 8.4–10.5) in the low-PNI group and 20.4 months (95% CI, 15.4–25.5) in the high-PNI group (Figure 2). The differences were statistically significant in all three subgroups (*p* < 0.05 for each).

**Figure 3:**
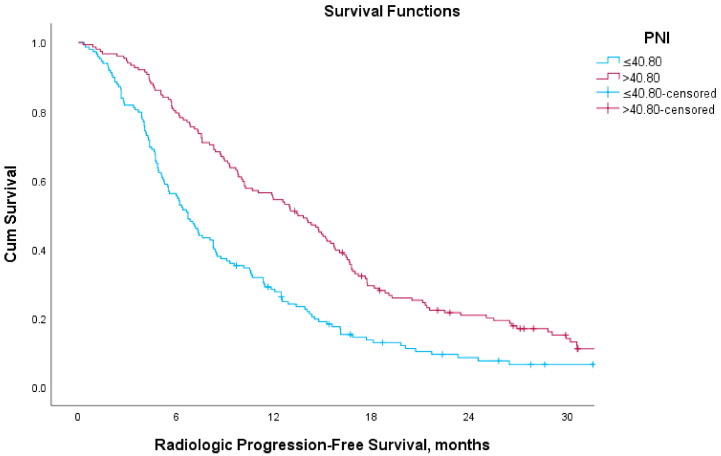
Kaplan–Meier curves for radiologic progression-free survival according to PNI.

Regarding rPFS, the median rPFS was 6.7 months (95% CI, 5.7–7.7) in the low-PNI group and 13.5 months (95% CI, 11.3–15.6) in the high-PNI group (Figure 3). In subgroup analyses, median rPFS in AA-treated patients was 6.1 months (95% CI, 4.3–8.0) versus 14.6 months (95% CI, 11.8–17.4) in low and high PNI groups, respectively. Among patients treated with ENZA, median rPFS was 7.4 months (95% CI, 5.0–9.8) in the low-PNI group and 15.1 months (95% CI, 11.4–18.7) in the high-PNI group. For those treated with CABA, median rPFS was 4.4 months (95% CI, 2.7–6.1) versus 9.0 months (95% CI, 6.5–11.4) in the low- and high-PNI groups, respectively (Figure 2). These differences were also statistically significant across all treatment subgroups (*p* < 0.05 for each).

PSA-PFS was approximately two-fold longer in the high-PNI group compared to the low-PNI group (10.2 vs. 5.1 months). This trend persisted within treatment subgroups: 9.8 vs. 4.6 months in the AA group, 12.9 vs. 7.3 months in the ENZA group, and 7.8 vs. 3.9 months in the CABA group.

### 3.4. Multivariate Cox Regression Analysis

Univariate Cox regression analysis identified several factors significantly associated with OS, including age, prostate surgery, radiotherapy to the primary tumor, anemia, neutrophilia, elevated alkaline phosphatase (ALP), elevated lactate dehydrogenase (LDH), visceral metastasis, bone metastasis, and PSA response (*p* < 0.05 for each). In multivariate analysis, high PNI (HR: 0.5; 95% CI: 0.33–0.70), PSA response (HR: 0.5; 95% CI: 0.33–0.65), elevated ALP (HR: 1.6; 95% CI: 1.11–2.28), and prior prostate surgery (HR: 0.6; 95% CI: 0.40–0.95) were identified as independent prognostic factors for OS (Table 3). 

Additionally, when PNI was analyzed as a continuous variable, it remained a significant predictor of OS, as shown in Supplementary Table S5.

### 3.5. Prognostic Model Based on Blood-Based Biomarkers

A prognostic model was developed using blood-based biomarkers that showed an association with OS in multivariate analysis at a significance level of *p* < 0.1. These included anemia, elevated ALP, and low PNI. The PSA response was excluded from the model due to its time-dependent nature and the fact that it is not available at treatment initiation. For each risk factor, a binary score was assigned: 1 point for the presence of the risk factor (anemia, elevated ALP, or low PNI), and 0 points for its absence. The cumulative risk score ranged from 0 to 3, and patients were classified into four prognostic groups accordingly: favorable (0 points), intermediate-low (1 point), intermediate-high (2 points), and poor (3 points).

**Figure 4:**
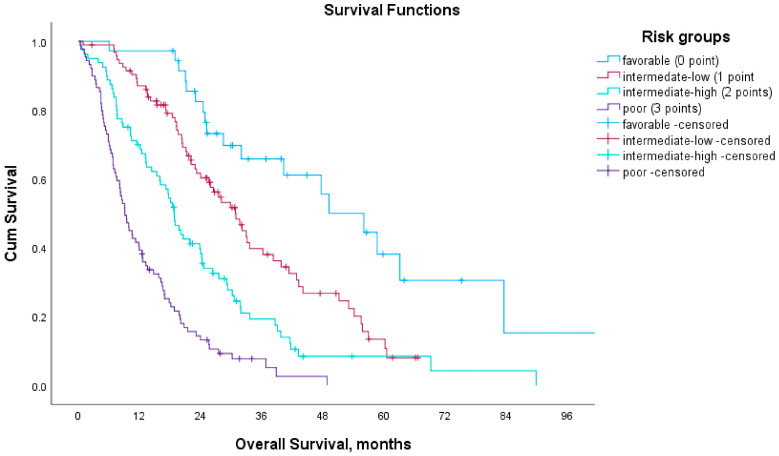
Kaplan–Meier curves for overall survival according to risk groups.

Based on this model, mOS differed significantly across prognostic risk groups. Patients in the favorable risk group (score 0) had a median OS of 49.1 months (95% CI, 33.9–64.4), whereas those in the intermediate-low (score 1), intermediate-high (score 2), and poor prognostic (score 3) groups had median OS durations of 30.8 months (95% CI, 25.2–36.3), 18.8 months (95% CI, 16.5–21.1), and 9.1 months (95% CI, 7.4–10.8), respectively (Table 4, Figure 4). Similarly, median PFS decreased steadily across these groups, with values of 18.2, 14.2, 8.4, and 5.2 months in the favorable, intermediate-low, intermediate-high, and poor prognostic groups, respectively.

## 4. Discussion

In this retrospective study, we demonstrated that the baseline PNI is an independent prognostic factor for both survival outcomes and treatment response in patients with mCRPC treated with AA, ENZA, or CABA. To our knowledge, this is the largest study conducted in a non-Asian population evaluating the prognostic significance of PNI in this setting. Low PNI was associated with poorer treatment response, shorter rPFS, and inferior OS across all treatment subgroups. Additionally, we proposed a simple blood-based prognostic model utilizing three readily available baseline laboratory parameters: PNI, ALP, and anemia status.

In prostate cancer, patients who initially respond to ADT eventually progress to a castration-resistant state. Several classes of agents—including taxanes, ARSIs, immunotherapy, radionuclide therapies, and PARP inhibitors in molecularly selected populations—have demonstrated survival benefits in the mCRPC setting [3]. In pivotal phase III trials, AA improved OS to 15.8 months in the post-chemotherapy setting and 34.7 months in the pre-chemotherapy setting [4,25]. In our study, where nearly 80% of AA-treated patients received the drug post-chemotherapy, median OS was 20.7 months, consistent with the literature. For ENZA, median OS was 18.4 and 35.3 months in the AFFIRM and PREVAIL studies for post- and pre-chemotherapy populations, respectively [5,26]. In our cohort, where approximately 65% of ENZA-treated patients had prior chemotherapy, mOS was 24.1 months. In the TROPIC study comparing CABA with mitoxantrone after DOCE, the mOS was 15.1 versus 12.7 months, favoring CABA [27]. Similarly, in our study, patients treated with CABA had a median OS of 16.1 months, despite some patients receiving intensive treatments.

Systemic inflammation and nutritional status are well-established contributors to cancer progression and prognosis [28,29,30,31]. Biomarkers such as NLR, PLR, and SII have been investigated as prognostic tools [11,12]. PNI combines serum albumin and lymphocyte count to reflect both nutritional and immune status. A low PNI may signify both malnutrition and impaired lymphocyte-mediated immune response, thereby contributing to poorer oncologic outcomes [22,23,32]. Since there is no universally accepted cut-off for PNI, previous studies have used either median values or ROC-derived thresholds. In our study, ROC analysis identified a cut-off of 41.26 (AUC: 0.643; sensitivity: 59.1%; specificity: 59.7%), but due to its moderate performance and proximity to the cohort’s median (40.8), we opted for the median value for group stratification. This also ensured consistency with prior literature.

Several studies in other malignancies—including lung, colorectal, esophageal, gynecologic, renal, and head and neck cancers—have found low PNI to be associated with decreased survival [15,16,17,18,19,20,21,33]. In prostate cancer, studies conducted across non-metastatic, hormone-sensitive metastatic, and castration-resistant metastatic settings have demonstrated the prognostic relevance of PNI; however, most of these studies have been limited to Asian populations and small sample sizes [22,23,32,34].

In mCRPC, there are a few studies with a low number of patients treated with AA and ENZA. A study by Fan et al., which included 112 mCRPC patients treated with AA, found that high PNI was associated with significantly improved OS and rPFS (28.4 vs. 18.4 months and 13.2 vs. 6.1 months, respectively) [10]. Similarly, Küçükarda et al. reported that in 101 patients treated with abiraterone or enzalutamide, both OS and rPFS were significantly longer in the high-PNI group (27.7 vs. 9.6 months and 13.3 vs. 4.2 months, respectively), with a possible survival advantage of enzalutamide over abiraterone among patients with low PNI [9]. Our findings, based on the largest mCRPC cohort from a non-Asian population to date, complement and extend these results. In our study, OS was nearly two-fold longer in high-PNI patients than in low-PNI patients (30.2 vs. 12.6 months), and this effect was consistently observed across AA, ENZA and CABA subgroups. Similar trends were seen for rPFS (13.5 vs. 6.7 months) and PSA-PFS (10.2 vs. 5.1 months), further supporting the utility of baseline PNI as a reliable, non-invasive biomarker for prognosis in mCRPC.

Studies on ENZA have demonstrated that patients with non-visceral disease, low-volume bone metastases, or lymph node-only involvement tend to have better survival outcomes [35]. In patients treated with AA after DOCE, poor prognostic factors have been identified, including elevated LDH and ALP levels, liver metastases, serum albumin ≤ 4 g/dL, ECOG performance status ≥ 2, and a time interval of ≤36 months from initiation of androgen deprivation therapy to AA treatment [36]. In our study, we developed a prognostic model incorporating blood-based biomarkers—PNI, ALP, and anemia status—due to their accessibility and clinical relevance. Based on this model, patients were stratified into four prognostic groups: favorable, intermediate-low, intermediate-high, and poor. This model is easy to calculate using routinely available laboratory tests and may help clinicians identify high-risk patients who could benefit from closer follow-up or intensified treatment strategies.

This study has several limitations. First, it was a retrospective, single-center analysis, which may introduce selection bias and limit the generalizability of the findings. Additionally, missing data for important clinical parameters such as ECOG performance status, comorbidities, and disease burden may have influenced the outcomes. Second, treatment heterogeneity was present—while some patients received AA or ENZA as first-line therapy, others received these agents in second or later lines, and all CABA patients were treated after docetaxel. This variability may have confounded survival outcomes. Third, the sample size of the CABA group was relatively small compared to the AA and ENZA groups, potentially limiting the power to detect subgroup differences. Lastly, the prognostic model developed using PNI, ALP, and anemia has not been externally validated, and its applicability should be confirmed in larger, prospective, and multi-center cohorts before clinical implementation.

## 5. Conclusions

In this study, we demonstrated that the PNI, a simple and readily accessible biomarker reflecting both nutritional and immunological status, is an independent predictor of treatment response and survival outcomes in patients with mCRPC treated with AA, ENZA, or CABA. Additionally, we developed a blood-based prognostic model incorporating PNI, ALP, and anemia status, which successfully stratified patients into distinct prognostic groups with significantly different survival outcomes. However, as this was a retrospective, single-center study with potential selection bias, treatment heterogeneity, and a relatively small cabazitaxel subgroup, our findings should be interpreted cautiously. If validated in prospective, multi-center studies, PNI and the proposed prognostic model may serve as practical tools for individual risk assessment and treatment decision-making in mCRPC.

## Figures and Tables

**Table 1:** Baseline clinicopathologic characteristics of the patients.

Characteristic	*n* = 299 (%)
Age of diagnosis (mean, SD)	65.2 (±9.0)
<70 yr	207 (69.2%)
≥70 yr	92 (30.8%)
PSA level ng/mL (median, IQR)	
(*n* = 186)	58.5 (16.8–180.8)
M1 disease at diagnosis	
Yes	179 (59.9%)
No	120 (40.1%)
Gleason score (*n* = 247)	
<8	52 (21.1%)
≥8	195 (78.9%)
Prostate Surgery	
Yes	61 (20.4%)
No	238 (79.6%)
Definitive or salvage radiotherapy	
Yes	81 (27.1%)
No	218 (72.9%)
Orchiectomy	
Yes	65 (21.7%)
No	234 (78.3%)
Comorbidities	
Hypertension	107 (35.8%)
Cardiovascular disease	82 (27.4%)
Diabetes	60 (20.1%)
Chronic kidney disease	22 (7.4%)
Previous docetaxel treatment	
Yes	234 (78.3%)
No	65 (21.7%)
Number of docetaxel cycles	
Median (range)	6 (1–32)
Treatment Subgroups	
Abiraterone acetate	133 (44.5%)
Enzalutamide	106 (35.5%)
Cabazitaxel	60 (20.0%)
PNI (median)	
PNI ≤ 40.8	148 (49.5%)
PNI > 40.8	151 (50.5%)

PSA, prostate-specific antigen; PNI, prognostic nutritional index. Note: Continuous variables are presented as mean ± standard deviation or median [interquartile range]; categorical variables as number (percentage).

**Table 2:** Association between PNI and clinicopathological parameters.

Parameter	Low PNI (PNI ≤ 40.8) *n* = 148 (%)	High PNI (PNI > 40.8) *n* = 151 (%)	*p*-Value
Abiraterone or Enzalutamide or Cabazitaxel starting age, years Mean (SD)	71.9 (±8.9)	68.3 (±9.4)	**<0.001**
Abiraterone or Enzalutamide or Cabazitaxel starting age			0.145
<70 years	64 (43.2%)	78 (51.7%)	
≥70 years	84 (56.8%)	73 (48.3%)	
PSA level-ng/mL (before treatment) (median/IQR)	70.1 (16.7–202.5)	24.7 (9.5–70.2)	**<0.001**
M1 disease at diagnosis			0.741
M0	58 (39.2%)	62 (41.1%)	
M1	90 (60.8%)	89 (58.9%)	
Gleason score			0.974
<8	26 (21.0%)	26 (21.1%)	
≥8	98 (79.0%)	97 (78.9%)	
Previous docetaxel treatment			0.056
Yes	39 (26.4%)	26 (17.2%)	
No	109 (73.6%)	125 (82.8%)	
Treatment groups Abiraterone Acetate Enzalutamide Cabazitaxel			0.682
	64 (43.2%)	69 (45.7%)	
	56 (37.8%)	50 (33.1%)	
	28 (18.9)	32 (21.2%)	
Metastasis sites			0.144
Bone			
Yes	131 (88.5%)	123 (82.6%)	
No	17 (11.5%)	26 (17.4%)	
Lymph nodes			0.581
Yes	94 (63.5%)	90 (60.4%)	
No	54 (36.5%)	59 (39.6%)	
Visceral			**0.023**
Yes	30 (20.3%)	16 (10.7%)	
No	118 (79.7%)	133 (89.3%)	
Hemoglobin level (g/dL)			**<0.001**
<13.5	134 (90.5%)	98 (64.9%)	
≥13.5	14 (9.5%)	53 (35.1%)	
Neutrophil level (x103/µL)			0.421
Low/Normal	115 (77.7%)	123 (81.5%)	
High	33 (22.3%)	28 (18.5%)	
Lymphocyte level (x103/µL)			**0.034**
Low	44 (29.7%)	29 (19.2%)	
Normal/High	104 (70.3%)	122 (80.8%)	
Albumin level			**<0.001**
<4 g/dL	126 (85.1%)	0 (0%)	
≥4 g/dL	22 (14.9%)	151 (100%)	
Alkaline phosphatase (ALP)			**<0.001**
Normal	58 (39.2%)	100 (66.2%)	
High (>120 U/L)	90 (60.8%)	51 (33.8%)	
LDH level			**0.001**
Normal	40 (36.0%)	64 (59.3%)	
High (>249 U/L)	71 (64.0%)	44 (40.7%)	

PNI, prognostic nutritional index; LDH, lactate dehydrogenase; PSA, prostate-specific antigen.

**Table 3:** Univariate and multivariate analyses of potential prognostic factors for overall survival.

Parameter	Univariate Analysis		Multivariate Analysis	
	HR (95% CI)	*p*-Value	HR (95% CI)	*p*-Value
Age (<70 vs. ≥70)	1.5 (1.15–2.02)	0.003	1.3 (0.90–1.88)	0.162
Prostate surgery	0.6 (0.41–0.85)	0.004	0.6 (0.40–0.95)	**0.029**
Prostate radiotherapy	0.7 (0.51–0.93)	0.017	1.2 (0.81–1.88)	0.321
Anemia	2.7 (1.93–3.92)	<0.001	1.4 (0.88–2.30)	0.149
Neutrophilia	1.4 (1.04–1.90)	0.027	1.3 (0.89–1.91)	0.169
ALP level (normal vs. > ULN)	2.5 (1.91–3.27)	<0.001	1.6 (1.11–2.28)	**0.012**
LDH level (normal vs. > ULN)	1.9 (1.42–2.65)	<0.001	1.3 (0.89–1.84)	0.185
Visceral metastasis	1.5 (1.04–2.12)	0.028	1.3 (0.77–2.04)	0.363
Bone metastasis	1.9 (1.31–2.89)	0.001	1.9 (1.08–3.35)	**0.025**
PSA response (absent vs. present)	0.4 (0.31–0.53)	<0.001	0.5 (0.33–0.65)	**<0.001**
PNI (low vs. high)	0.4 (0.29–0.49)	<0.001	0.5 (0.33–0.70)	**<0.001**

HR, hazard ratio; CI, confidence interval; ALP, alkalen phosphatase; LDH, lactate dehydrogenase; PSA, prostate-specific antigen; PNI, prognostic nutritional index.

**Table 4:** Survival data in prognostic groups according to the prognostic tool.

Score	Prognosis	mOS (Months)	mPFS (Months)
0	Favorable	49.1	18.2
1	Intermediate-low	30.8	14.2
2	Intermediate-high	18.8	8.4
3	Poor	9.1	5.2

mOS, median overall survival; mPFS, median progression-free survival.

## Data Availability

The datasets used and/or analyzed during the current study available from the corresponding author on reasonable request.

## Supplementary Materials

The following supporting information can be downloaded at: https://www.mdpi.com/article/10.3390/medicina61061105/s1, Table S1: Baseline clinicopathologic characteristics of the treatment subgroups; Table S2: Pre-treatment clinicopathological characteristics according to treatment subgroups; Table S3: Overall survival outcomes according to treatment subgroup and docetaxel treatment sequence; Table S4. Radiologic progression-free survival outcomes according to treatment subgroup and docetaxel treatment sequence; Table S5: Cox regression analysis for prognostic nutritional index as a continuous variable.

## Abbreviations

Abiraterone acetate; ADT: Androgen deprivation therapy; ALP: Alkaline phosphatase; AR: Androgen receptor; ARSIs: Androgen-receptor signaling inhibitors; CABA: Cabazitaxel; CI: Confidence interval; DOCE: Docetaxel; ENZA: Enzalutamide; HR: Hazard Ratio; LDH: Lactate dehydrogenase; mCRPC: Metastatic castration-resistant prostate cancer; NLR: Neutrophil lymphocyte ratio; OS: Overall survival; PARP: Poly (ADP-ribose) polymerase; PLR: Platelet lymphocyte ratio; PNI: Prognostic nutritional index; PSA: Prostate specific antigen; PFS: Progression-free survival; rPFS: Radiographic progression-free survival; ROC: Receiver operating characteristic; SII: Systemic immune inflammatory index.
