# Supplementary material for: Prognostic Nutritional Index and a Blood-Based Prognostic Tool in Prostate Cancer Treated with Abiraterone, Enzalutamide or Cabazitaxel
Source: Medicina (Kaunas). 2025 Jun 18;61(6):1105. doi: 10.3390/medicina61061105 (PMC12195598; doi:10.3390/medicina61061105)
Supplement: Supplementary file 1 [file medicina-61-01105-s001.zip › medicina-3653318-supplementary.pdf]

Supplementary Tables

Table S1. Baseline clinicopathologic characteristics of the treatment subgroups

Characteristic	Total n = 299 (%)	Abiraterone Acetate Subgroup n=133	Enzalutamide Subgroup n=106	Cabazitaxel Subgroup n=60
**Age of diagnosis (mean, SD)**	65.2 (± 9.0)	66.8 (± 9.2)	66.3 (± 8.5)	59.6 (± 7.4)
**<70 yr**	207 (69.2%)			
**≥70 yr**	92 (30.8%)			
**M1 disease at diagnosis**				
**Yes**	179 (59.9%)	76 (57.1%)	61 (57.5%)	42 (70.0%)
**No**	120 (40.1%)	57 (42.9%)	45 (42.5%)	18 (30.0%)
**Gleason score (n=247)**				
**<8**	52 (21.1%)	29 (27.6%)	12 (14.1%)	11 (19.3%)
**≥8**	195 (78.9%)	76 (72.4%)	73 (85.9%)	46 (80.7%)
**Prostate Surgery**				
**Yes**	61 (20.4%)	29 (21.8%)	26 (24.5%)	6 (10.0%)
**No**	238 (79.6%)	104 (78.2%)	80 (75.5%)	54 (90.0%)
**Definitive or salvage radiotherapy**				
**Yes**	81 (27.1%)	33 (24.8%)	35 (33.0%)	13 (21.7%)
**No**	218 (72.9%)	100 (75.2%)	71 (67.0%)	47 (78.3%)
**Orchiectomy**				
**Yes**	65 (21.7%)	21 (15.8%)	12 (11.3%)	6 (10.0%)
**No**	234 (78.3%)	112 (84.2%)	94 (88.7%)	54 (90.0%)
**Comorbidities**				
**Hypertension**	107 (35.8%)	52 (39.1%)	43 (40.6%)	12 (20.0%)
**Cardiovascular disease**	82 (27.4%)	37 (27.8%)	33 (31.1%)	12 (20.0%)
**Diabetes**	60 (20.1%)	24 (18.0%)	28 (26.4%)	8 (13.3%)
**Chronic kidney disease**	22 (7.4%)	9 (6.8%)	9 (8.5%)	4 (6.7%)
**Previous docetaxel treatment**				
**Yes**	234 (78.3%)	106 (79.7%)	68 (64.2%)	60 (100.0 %)
**No**	65 (21.7%)	27 (20.3%)	38 (35.8%)	0
**Number of docetaxel cycles Median (range)**	6 (1-32)	6 (1-32)	6 (1-16)	8 (3-32)
**PNI (median)**				
**PNI ≤ 40.8**	148 (49.5%)	64 (48.1%)	56 (52.8%)	28 (46.7%)
**PNI > 40.8**	151 (50.5%)	69 (51.9%)	50 (47.2)	32 (53.3%)

PSA, prostate-specific antigen; PNI, prognostic nutritional index
Note: Continuous variables are presented as mean ± standard deviation or median [interquartile range]; categorical variables as number (percentage).

Table S2. Pre-treatment clinicopathological characteristics according to treatment subgroups

Characteristic	Total n = 299 (%)	Abiraterone Asetate Subgroup n=133	Enzalutamide Subgroup n=106	Cabazitaxel Subgroup n=60
**Abiraterone or Enzalutamide or Cabazitaxel starting age, years Mean (SD)**	70.1 (± 9.3)	72.2 (±8.9)	70.7 (± 9.1)	64.2 (± 8.1)
**Abiraterone or Enzalutamide or Cabazitaxel starting age**				
**< 70 years**	142 (47.5%)	52 (39.1%)	45 (42.5%)	45 (75.0%)
**≥ 70 years**	157 (52.5%)	81 (60.9%)	61 (57.5%)	15 (15.0%)
**PSA level-ng/ml (before treatment) (median/IQR)**	36 (10.7 – 106.9)	39.8 (15.6 – 126.0)	16.0 (5.4 – 44.2)	85.0 (41.4 – 199.0)
**Metastasis sites**				
**Bone**				
**Yes**	254 (85.5%)	112 (14.5%)	90 (84.9%)	52 (86.7%)
**No**	43 (14.5%)	19 (85.5%)	16 (15.1%)	8 (13.3%)
**Lymph nodes**				
**Yes**	184 (62.0%)	64 (48.9%)	75 (70.8%)	45 (75.0%)
**No**	113 (38.0%)	67 (51.1%)	31 (29.2%)	15 (15.0%)
**Visceral**				
**Yes**	46 (15.5%)	8 (6.1%)	21 (19.8%)	17 (28.3%)
**No**	251 (84.5%)	123 (93.9%)	85 (80.2%)	43 (71.7%)
**Hemoglobin level (g/dl)**				
**< 13.5**	232 (77.6%)	104 (78.2%)	77 (72.6%)	51 (85.0%)
**≥13.5**	67 (22.4%)	29 (21.8%)	29 (27.4%)	9 (15.0%)
**Neutrophil level (x103/µL)**				
**Low/Normal**	238 (79.6%)	105 (78.9%)	92 (86.8%)	41 (68.3%)
**High**	61 (20.4%)	28 (21.1%)	14 (13.2%)	19 (31.7%)
**Lymphocyte level (x103/µL)**				
**Low**	73 (24.4%)	30 (22.6%)	30 (28.3%)	13 (21.7%)
**Normal/High**	226 (75.6%)	103 (77.4%)	76 (71.7 %)	47 (78.3%)

**Albumin level**				
**< 4 g/dl**	126 (42.1%)	58 (43.6%)	47 (44.3%)	21 (35.0%)
**≥ 4 g/dl**	173 (57.9%)	75 (56.4%)	59 (55.7%)	39 (65.0%)
**Alkaline phosphatase (ALP)**				
**Normal**	158 (52.8%)	68 (51.1%)	69 (65.1%)	21 (35.0%)
**High (> 120 U/L)**	141 (47.2%)	65 (48.9%)	37 (34.9%)	39 (65.0%)
**LDH level**				
**Normal**	104 (47.5%)	47 (47.0%)	51 (59.3%)	6 (18.2%)
**High (> 249 U/L)**	115 (52.5%)	53 (53.0%)	35 (40.7%)	27 (81.8%)

PSA, prostate-specific antigen; PNI, prognostic nutritional index
Note: Continuous variables are presented as mean ± standard deviation or median [interquartile range]; categorical variables as number (percentage).

Table S3. Overall survival outcomes according to treatment subgroup and docetaxel treatment sequence

Treatment Group	Docetaxel Sequence	Median Survival (months)	95% CI for Mean (months)	p value
Abiraterone Acetate	Pre-docetaxel	23.9 ± 7.9	8.4 – 39.4	0.74
	Post-dosetaxel	20.2 ± 1.9	16.4 – 23.9	
Enzalutamide	Pre-docetaxel	19.71 ± 5.43	9.1 – 30.4	0.36
	Post-docetaxel	25.59 ± 1.57	22.5 – 28.7	
Cabazitaxel	Post-docetaxel	16.1 ± 3.4	9.4 – 22.8	

Table S4. Radiologic progression-free survival outcomes according to treatment subgroup and docetaxel treatment sequence

Treatment Group	Docetaxel Sequence	Median Survival (months)	95% CI for Mean (months)	p value
Abiraterone Acetate	Pre-docetaxel	9.3 ± 4.2	1.1 – 17.4	0.21
	Post-docetaxel	10.1 ± 1.5	7.1 – 12.9	
Enzalutamide	Pre-docetaxel	11.4 ± 5.2	1.2 – 21.6	0.95
	Post-docetaxel	11.3 ± 1.8	7.8 – 14.9	
Cabazitaxel	Post-docetaxel	7.5 ± 0.8	6.0 – 9.1	

Table S5. Cox regression analysis for prognostic nutritional index as a continuous variable

**Analysis**	**HR (95% CI)**	**p-value**
Univariate	0.919 (0.898 – 0.941	<0.001
Multivariate	0.915 (0.874 – 0.958)	<0.001
